# Advanced age and long-term care facility stay as risk factors for methicillin-resistant Staphylococcus aureus infection

**DOI:** 10.1186/2047-2994-4-S1-P191

**Published:** 2015-06-16

**Authors:** M Pobiega, A Chmielarczyk, M Pomorska-Wesołowska, G Ziolkowski, J Wojkowska-Mach

**Affiliations:** 1 Jagiellonian University Medical School, Kraków, Poland; 2 Higher School of Medicine in Sosnowiec, Sosnowiec, Poland

## Introduction

Methicillin-resistant *Staphylococcus aureus* (MRSA) is no longer only a nosocomial pathogen. It has emerged as an important cause of community-associated infections.

## Objectives

The aim of this study was to analyze risk factors for MRSA infections in geriatric population (60 years and more) in south of Poland and assess antimicrobial susceptibility of the isolates.

## Methods

Non-repetitive samples were collected from hospitalized (12 hospitals) and non-hospitalized (outpatient care and three long-term care facilities) patients presenting infections (wounds, lower respiratory infection (LRI), bloodstream and eye infections) throughout the southern Poland (Malopolska and Silesia) in 2013. Relation between age, type of infection, presence of comorbidities and probability and epidemiology of MRSA infection were analyzed.

## Results

MRSA prevalence was 17.2%, in patients with advanced age (>90 years) was 42.1% and in LRI was 39.3%. Factors association with MRSA-infection in geriatric population were advanced age, (OR 2.78; 95%CI 1.079-7.163), the presence of lower respiratory tract infection (OR 3.44; 95%CI 1.544-7.644) and staying in the LTCF (OR 5.27; 95%CI 2.02-13.74). Community-acquired infections (prevalence 13.9%) were significantly more often connected with MSSA, than MRSA (OR 0.42; 95%CI 0.24-0.68). MRSA isolates were more often resistant for all studied antibiotics, except teicoplanin and oxazolidinones. MIC50 and MIC90 for vancomicin and tigecycline were higher for MRSA strains.

## Conclusion

Age 90+ and LTCF staying are important risk factors for MRSA - increased risk of drugresistance almost 3-times. High drug resistance indicates a significant therapeutic limitations, especially in the elderly.

## Disclosure of interest

None declared.

